# The tubulin polymerization inhibitor gambogenic acid induces myelodysplastic syndrome cell apoptosis through upregulation of Fas expression mediated by the NF-κB signaling pathway

**DOI:** 10.1080/15384047.2024.2427374

**Published:** 2024-11-14

**Authors:** Cheng Zhong, Shijun Wang, Lei Xia, Xiaoman Yang, Liguang Fang, Xianyi Zhang, Mengyue Wang, Haijun Zhao, Guanghui Wang, Jinglong Wu, Ruijian Guo, Ming Zhong, Eiichi Gohda

**Affiliations:** a Department of Pathology, College of Traditional Chinese Medicine, Shandong University of Traditional Chinese Medicine, Jinan, China; b Postdoctoral Research Workstation of Integrated Traditional Chinese and Western Medicine, Shandong University of Traditional Chinese Medicine, Jinan, China; c Division of Stem Cell and Molecular Medicine, Center for Stem Cell Biology and Regenerative Medicine, The Institute of Medical Science, The University of Tokyo, Tokyo, Japan; d Department of Pharmacology and Toxicology, School of Pharmacy, Jining Medical University, Rizhao, China; e Graduate School of Interdisciplinary Science and Engineering in Health Systems, Okayama University, Okayama, Japan; f Department of Pharmaceutics, College of pharmacy, Nanjing University of Chinese Medicine, Nanjing, China; g Division of Pharmaceutical Sciences, Okayama University Graduate School of Medicine, Dentistry and Pharmaceutical Sciences, Okayama, Japan

**Keywords:** Gambogenic acid, apoptosis, NF-κB, Fas, microtubule, G2/M arrest, MCL-1, myelodysplastic syndrome

## Abstract

The development of an effective treatment for myelodysplastic syndrome (MDS) is needed due to the insufficient efficacy of current therapies. Gambogenic acid (GNA) is a xanthone constituent of gamboge, a resin secreted by *Garcinia hanburyi* Hook. f. GNA exhibits antitumor and apoptosis-inducing activities against some cancer cells, but the mechanism is unknown. This study aimed to validate the anti-proliferative and apoptosis-inducing effects of GNA on MDS cells and to elucidate the mechanisms underlying those activities. Apoptosis, proliferation and cell cycle of MDS-L cells were assessed by the caspase 3/7 assay, cell counting and flow cytometry, respectively. The levels of apoptotic, tubulin, NF-κB pathways, and Fas proteins were determined by Western blotting. CRISPR/Cas9 knockout (KO) plasmids were used to generate KO cells of p65 and Fas. MDS cell growth in a xenograft model was evaluated by the AkaBLI system. GNA induced MDS cell apoptosis, accompanied by a reduction in the anti-apoptotic protein MCL-1 expression, and inhibited their growth in vitro and in vivo. GNA combined with the MCL-1 inhibitor MIK665 potently suppressed the proliferation of MDS cells. GNA interfered with tubulin polymerization, resulting in G2/M arrest. GNA induced NF-κB activation and upregulation of Fas, the latter of which was inhibited by p65 KO. GNA-induced apoptosis was attenuated in either p65 KO or Fas KO cells. These results demonstrate that GNA inhibited tubulin polymerization and induced apoptosis of MDS cells through upregulation of Fas expression mediated by the NF-κB signaling pathway, suggesting a chemotherapeutic strategy for MDS by microtubule dynamics disruption.

## Introduction

Myelodysplastic syndrome (MDS) is an abnormal malignant myeloid clonal disease characterized by hematopoietic failure that may result in clone evolution to acute myeloid leukemia (AML).^1^ Studies have shown that the pathology of MDS is very complicated. Several factors such as aging, radiation and hazardous chemicals may lead to changes of the bone marrow microenvironment, somatic gene mutations and epigenetic changes, in turn leading to increased cell apoptosis or dedifferentiation of progenitor cells, which may be transformed into AML. The pathogenesis of MDS is related to various factors such as cytogenetics, immune system and bone marrow hematopoietic microenvironment, which may contribute to this multi-stage pathological process.^2^

The risk of MDS is evaluated by the International Prognostic Scoring System (IPSS).^3^ The most common symptom of low-risk MDS is anemia, and erythropoiesis-stimulating agents are therefore used as a first-line treatment. For high-risk MDS patients, allogeneic hematopoietic stem cell transplantation (ALLO-HSCT) is an important therapeutic strategy, but only a small number of MDS patients can receive ALLO-HSCT because of age, comorbidities, and lack of donors, and poor survival after ALLO-HSCT was reported for patients with chromosome 7 abnormal MDS.^4^ Therefore, chemotherapy becomes another choice for patients who are unable to receive transplantation. Currently, demethylating agents (hypomethylating agents, HMAs) are the first-line drugs for treatment of high-risk MDS. HMAs may be incorporated into DNA and cause covalent binding with DNA methyltransferase, which results in DNA hypomethylation and cytotoxicity. Inhibition of DNA methylation has extended the overall survival of patients with MDS and patients with AML transformed from MDS.^5^ However, less than 50% of patients have responded to HMA treatment, and most patients have a short survival period with only a few of them achieving long-lasting remission.^6^ Therefore, the development of a more effective therapeutic strategy for MDS treatment is important.

Natural products have been important sources of new drugs, especially anticancer drugs and anti-infective agents, for which 25% and 27% of the drugs, respectively, have been of natural origin including semisynthetic derivatives of natural products over the last four decades.^7^ Gamboge, a dry resin secreted by *Garcinia hanburyi* Hook. f., has traditionally been used as emetic, laxative and parasiticide and for the treatment of cancer, infected wound and edema.^8–10^ Recent studies have shown that gamboge has a potent anticancer activity against certain solid tumors^11^ and contains the xanthone gambogenic acid (GNA) as an active constituents.^12^ GNA has a broad range of anti-tumor effects with a low level of toxicity in normal cells compared with that in cancer cells.^12,13^ It has also been shown to induce apoptosis in different types of cancer cells,^14,15^ but little is known about the mechanism. Furthermore, there is no information concerning the sensitivity of MDS cell growth and apoptosis to GNA.

In this report, we describe the significant anti-proliferative and apoptosis-inducing effects of GNA against MDS cells and its molecular mechanism. Our results showed that GNA inhibited tubulin polymerization and caused cell cycle arrest at the G2/M phase in addition to the G0/G1 phase arrest. Moreover, GNA induced apoptosis of MDS cells, accompanied by a decrease in MCL-1 level, through upregulation of Fas expression mediated by the NF-κB signaling pathway. Our results suggest a chemotherapeutic strategy for MDS by disruption of microtubule dynamics.

## Results

### Apoptosis and growth inhibition of MDS cells induced by GNA

**Figure 1.**
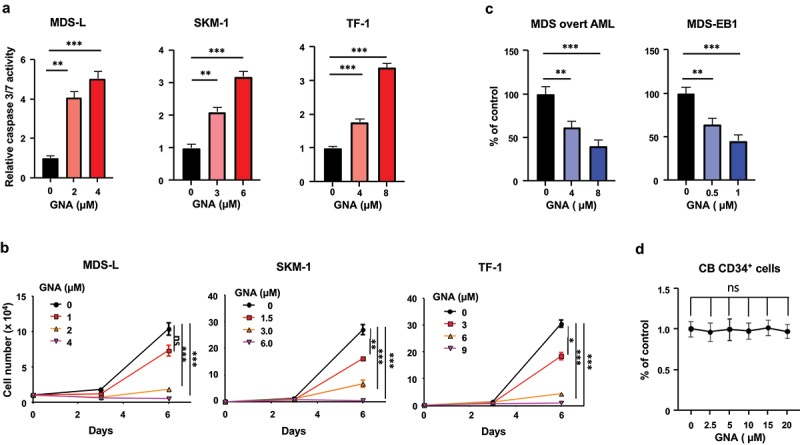
Apoptosis and growth inhibition of MDS cells induced by GNA. (a) Caspase 3/7 activities in gna-treated cells. Three cell lines (MDS-L, SKM-1, and TF-1) were incubated with the indicated doses of GNA for 24 h. (b) Cell growth after GNA treatment. The three cell lines (MDS-L, SKM-1, and TF-1) were incubated with the indicated doses of GNA, and the number of viable cells was counted at days 3 and 6. (c) Growth of primary MDS cells treated with GNA. Two different types of CD34^+^ MDS cells (MDS overt AML and MDS-EB1) were isolated and cultured for 48 h with the indicated doses of GNA. Cell growth was determined by the MTS assay. (d) Growth of CB CD34^+^ cells treated with GNA. CB CD34^+^ cells were cultured for 72 h with the indicated doses of GNA. Cell growth was determined by the MTS assay. Data are shown as means ± SD (*n* = 3). *p < .05, **p < .01 and ***p < .001; ns: not-significant (one-way ANOVA followed by Tukey’s multiple comparison test).

We examined whether GNA induces MDS cell apoptosis and suppression of cell proliferation. Two types of MDS cell lines, MDS-L and SKM-1, were used in this in vitro test: MDS-L is a subline established from the human MDS cell line,^16,17^ and SKM-1 is a cell line established from an MDS/AML patient with no chromosomal abnormalities, but it has point mutations in *NRAS* and *KRAS*.^18^ TF-1 is an immortal cell line derived from human erythroleukemia.^19^ Since the effector caspases-3 and/or -7 are activated and proteolytically cleave various cellular targets, leading to cell apoptosis,^20^ we determined caspase 3/7 activity using a luminogenic substrate. GNA increased caspase 3/7 activity in all of the tested cell lines including MDS-L, SKM-1 and TF-1 (Figure 1a). Percentages of dead cells were 44.4 ± 8.1 (*p* < .001 vs. control, 4.9 ± 2.2), 40.7 ± 6.3 (*p* < .001 vs. control, 3.4 ± 1.0) and 53.2 ± 5.5 (*p* < .001 vs. control, 4.6 ± 0.9) in MDS-L, SKM-1 and TF-1 cell cultures treated with 4, 6 and 8 μM GNA, respectively, as determined by a trypan blue exclusion test. GNA also inhibited the proliferation of all cell lines (Figure 1b) and the proliferation of primary MDS cells, MDS-EB1 and MDS overt AML (Figure 1c), as assessed by viable cell counting and a formazan formation assay, respectively. However, human cord blood (CB) CD34^+^ cells were more tolerant to the chemotherapeutic GNA (Figure 1d).

### GNA-induced inhibition of MCL-1 expression and sensitization of MDS cells to the MCL-1 inhibitor

**Figure 2.**
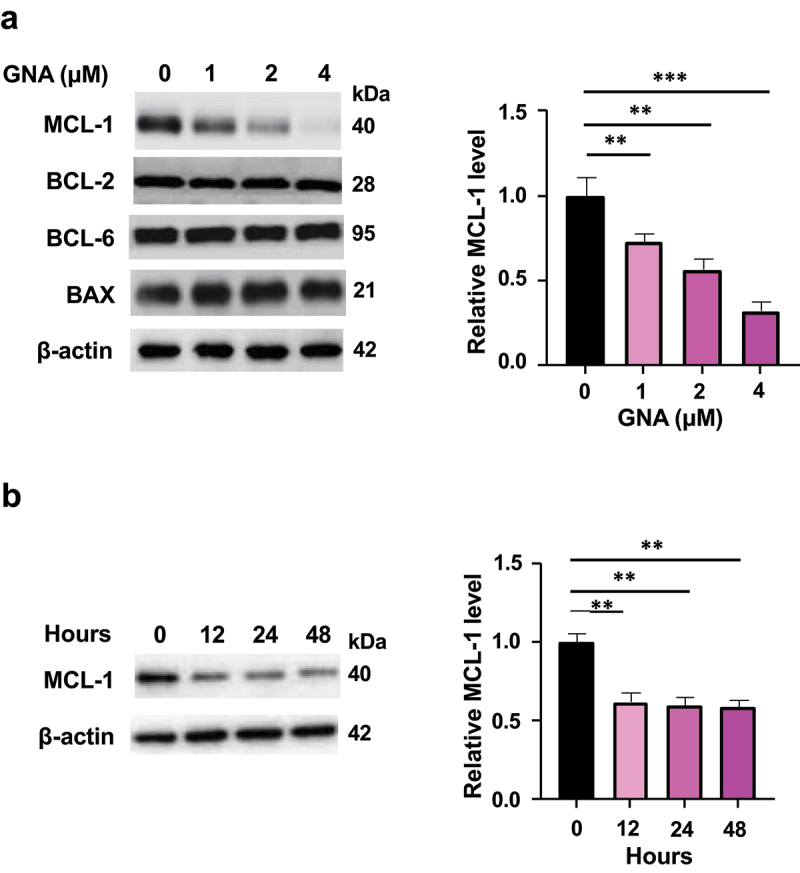
GNA inhibits MCL-1 expression. (a) Expression of BCL-2 family proteins and BCL-6 in MDS-L cells after 24-h treatment with 1, 2 or 4 μM GNA. Intensity of the signals on immunoblots was analyzed and normalized to that of β-actin by image lab (bio-rad) and expressed as fold change to the level of untreated cells. (b) MCL-1 expression in MDS-L cells treated with 2 μM GNA for the indicated hours. Data are shown as means ± SD (*n* = 3). **p < .01 and ***p < .001 (one-way ANOVA followed by Tukey’s multiple comparison test).

**Figure 3.**
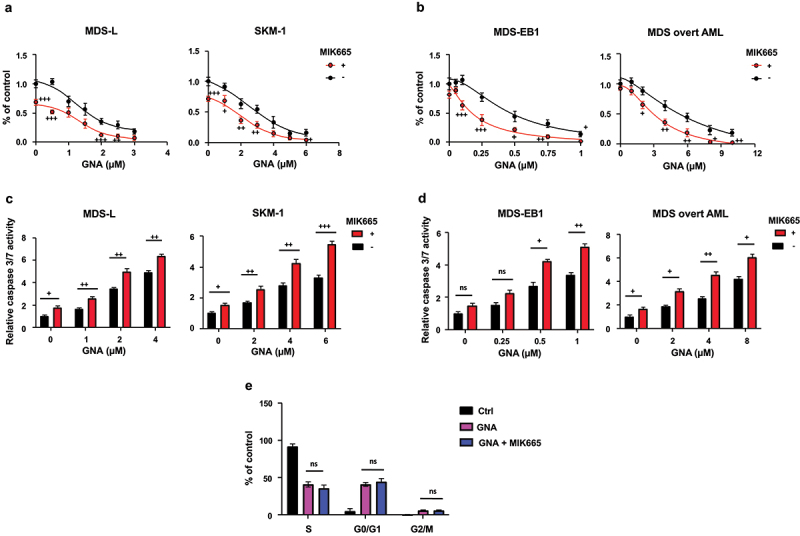
GNA sensitizes MDS cells to the MCL-1 inhibitor MIK665. (a, b) MTS viability curves of MDS-L, SKM-1, MDS-EB1, and MDS overt AML cells treated with the indicated doses of GNA alone or in combination with 50 nM MIK665 (selleckchem #S8836) for 24 h (a) or 48 h (b). (c, d) Caspase 3/7 activities after 24-h (c) or 48-h (d) treatment with the indicated doses of GNA or the combination of GNA and 50 nM MIK665. (e) Cell cycles of MDS-L cells treated with the combination of 2 μM GNA and 50 nM MIK665 for 24 h and then exposed to BrdU for 4 h before analysis. Data are shown as means ± SD (*n* = 3). (a-d) ^+^*p* < .05, ^++^*p* < .01, ^+++^*p* < .001, and ns (not-significant) vs. GNA alone (student’s t-test). (e) ns (one-way ANOVA followed by Tukey’s multiple comparison test).

Since GNA induced MDS cell apoptosis, we measured the expression levels of the pro-apoptotic protein BAX, the anti-apoptotic proteins BCL-2 and MCL-1, and the transcriptional repressor BCL-6, which suppresses the expression of cell death genes. The expression of MCL-1 was reduced in a dose-dependent but time-independent manner by GNA, whereas the expression of BCL-2, BCL-6 and BAX was not changed by GNA (Figure 2a,b). We then determined the combined effect of GNA and the MCL-1 inhibitor MIK665 (S64315).^21^ As compared with GNA alone, the combination led to a further suppression of cell proliferation (Figure 3a,b) and a further increase of caspase 3/7 activity (Figure 3c,d) in MDS-L, SKM-1, MDS-EB1, and MDS overt AML cells. Three μM GNA plus 50 nM MIK665, 6 μM GNA plus 50 nM MIK665, 1 μM GNA plus 50 nM MIK665, and 10 μM GNA plus 50 nM MIK665 almost completely inhibited the growth of MDS-L, SKM-1, MDS-EB1, and MDS overt AML cells, respectively (Figure 3a,b).

### Cell cycle arrest induced by GNA

**Figure 4.**
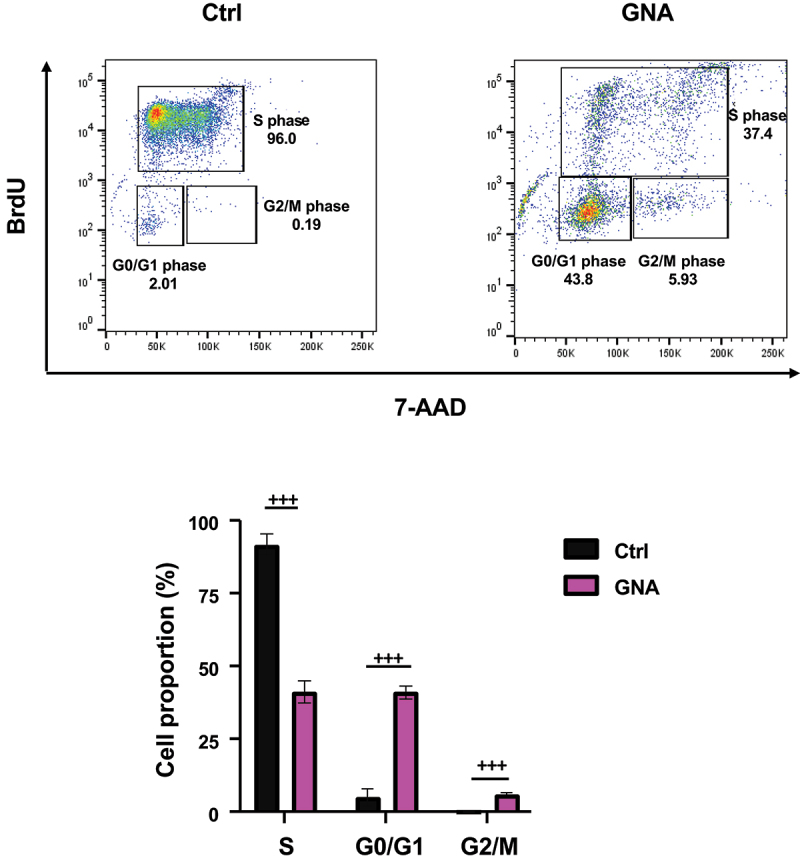
Cell cycle arrest induced by GNA. MDS-L cells was incubated with 2 μM GNA for 24 h and then exposed to BrdU for 4 h before analysis. Representative density plots of BrdU incorporation (y-axis) versus DNA content assessed by 7-AAD staining (x-axis) are shown in the top panel. The order of highest density of events within the cell population to lowest is: red, yellow, green, and blue. The proportion of cells in the indicated phase of the cell cycle is shown as means ± SD (*n* = 3) in the bottom panel. ^+++^*p* < .001 (student’s t-test).

To elucidate the molecular mechanism responsible for the inhibition of cell growth, cell cycle progression of GNA-treated MDS-L cells was determined by flow cytometric analysis using bromodeoxyuridine (BrdU) incorporation into DNA and 7-aminoactinomycin D (7-AAD) staining of DNA. As shown in Figure 4, the percentages of G0/G1 and G2/M phase cells increased with a concomitant decrease in the percentage of S phase cells by GNA treatment, the increase in the percentage of G2/M phase cells being smaller than that in the percentage of G0/G1 phase cells. Thus, GNA induced both G0/G1 arrest and G2/M arrest of MDS-L cells. By contrast with the further inhibitory effect of combined GNA and MIK665 on MDS-L cell proliferation (Figure 3a), the percentages of G0/G1 and G2/M phase cells did not further increase by their combination as compared with those of cells treated with GNA alone (Figure 3e).

### Inhibition of tubulin polymerization by GNA

**Figure 5.**
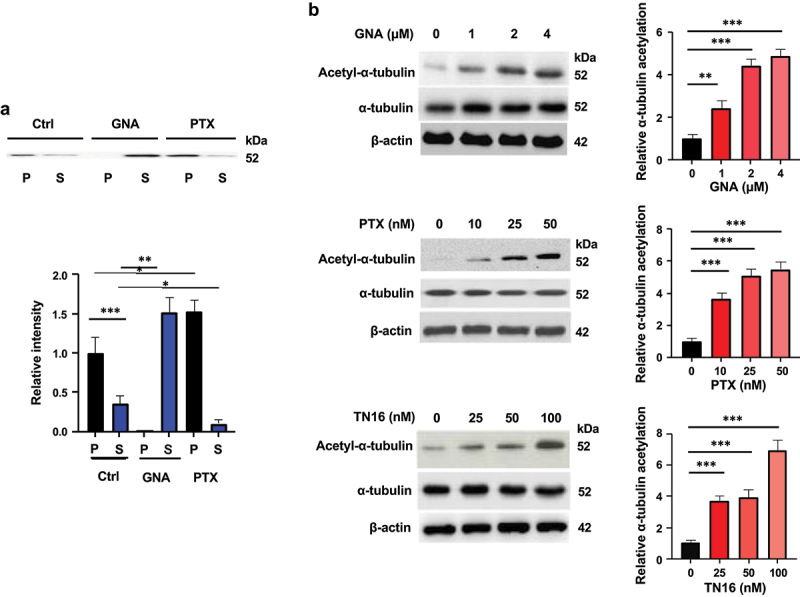
GNA inhibits tubulin polymerization and increases tubulin acetylation in MDS cells. (a) Tubulin contents in polymerized (P) vs. soluble (S) fractions of gna-treated MDS-L cells. MDS-L cells were treated with 10 μM of GNA or 1 μM paclitaxel (PTX) for 6 h, and 5 × 10^5^ cells were taken from each sample. The fractions containing soluble and polymerized tubulin were collected, separated by SDS-PAGE, and subjected to immunoblotting with an anti-α-tubulin antibody. Band intensity was analyzed by image lab, expressed as fold change to the level of control polymerized fraction, and is shown as means ± SD (*n* = 3). (b) Tubulin acetylation of MDS-L cells treated with the indicated concentrations of GNA, PTX and TN16 for 6 h. Tubulin acetylation was assessed by Western blotting using an anti-acetyl-α-tubulin antibody. Band intensity was analyzed and normalized to that of α-tubulin by image lab, expressed as fold change to the level of untreated cells, and is shown as means ± SD (*n* = 3). *p < .05, **p < .01 and ***p < .001 (one-way ANOVA followed by Tukey’s multiple comparison test).

A dynamic balance of microtubules between polymerization and depolymerization plays a central role in cell mitosis. Hence, microtubule-targeting (stabilizing or destabilizing) agents block cell mitosis in the G2/M phase. Microtubules are formed by the polymerization of a dimer of two globular proteins, α-tubulin and β-tubulin. Effects of GNA on the levels of soluble (depolymerized) tubulin versus polymerized tubulin in MDS-L cells were determined. Cells were cultured with or without GNA (10 μM) and paclitaxel (1 μM) for 6 hours, and the cells in the permeabilizing buffer were then separated into supernatant and polymerized tubulin fractions via centrifugation. Visualization of tubulin fractions by Western blotting demonstrated that the GNA treatment resulted in an increase of depolymerized microtubules, indicating inhibition of tubulin polymerization (Figure 5a). In contrast, polymerized microtubules increased in cells treated with paclitaxel (Figure 5a), which stabilizes microtubules against depolymerization.^22^

It has been reported that microtubule-targeting agents including paclitaxel and TN16 increase acetylation of α-tubulin.^23,24^ Thus, we determined microtubule acetylation and found increased levels of acetylated tubulin in MDS-L cells treated with GNA as well as in the cells treated with paclitaxel and TN16 (Figure 5b).

**Figure 6.**
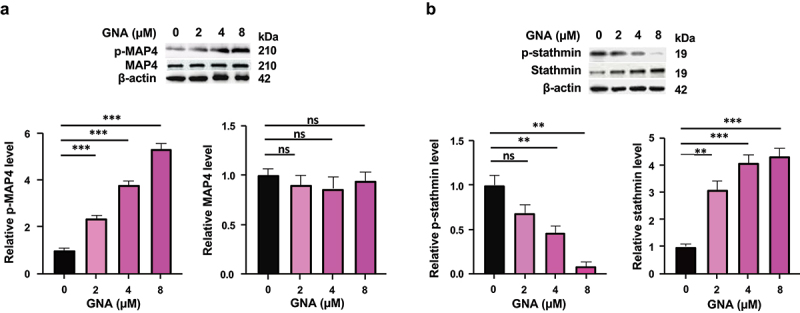
Expression and phosphorylation of MAP4 (a) and stathmin (b) in MDS-L cells treated with the indicated doses of GNA for 24 h. Band intensity was analyzed and normalized to that of β-actin by image lab and expressed as fold change to the level of untreated cells. Data are shown as means ± SD (*n* = 3). **p < .01 and ***p < .001; ns: not-significant (one-way ANOVA followed by Tukey’s multiple comparison test).

Microtubule assembly is influenced by many regulatory proteins including microtubule-binding proteins.^25,26^ We selected two microtubule-binding proteins: microtubule-associated protein 4 (MAP4) categorized as stabilizers and stathmin categorized as destabilizers to examine the effect of GNA.^25^ Microtubule-stabilizing activity of MAP4 is reduced by phosphorylation of Ser787.^27^ Phosphorylation of stathmin on Ser63 reduces its microtubule-destabilizing activity.^28^ As shown in Figure 6, GNA dose-dependently upregulated phosphorylation of MAP4 with constant MAP4 levels, whereas phosphorylation of stathmin was dose-dependently downregulated by GNA with a concomitant increase in the level of stathmin.

### Involvement of NF-κB activation in GNA-induced apoptosis

**Figure 7.**
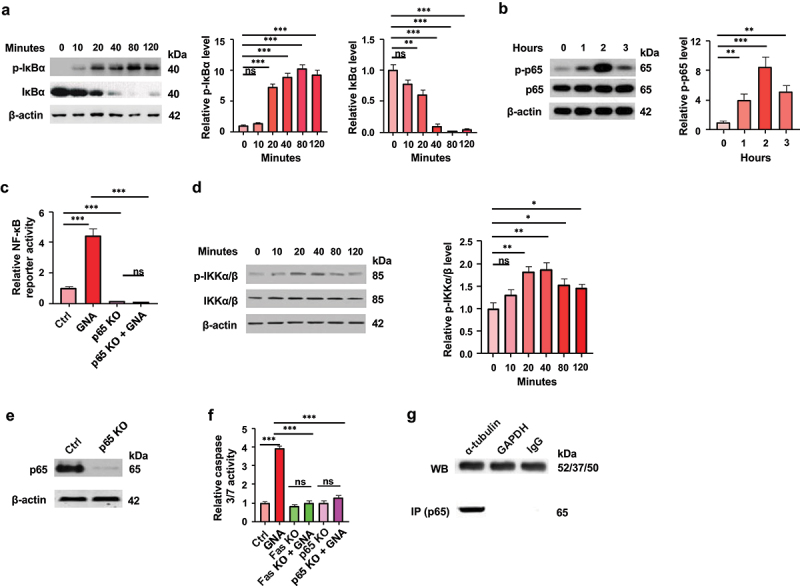
Involvement of nf-κB activation and fas expression in gna-induced apoptosis of MDS-L cells. (a) Phosphorylation (Ser32) and degradation of IκBα in MDS-L cells treated with 2 μM GNA for the indicated times. Band intensity was analyzed and normalized to that of β-actin by image lab and expressed as fold change to the level of untreated cells. (b) Phosphorylation (Ser536) of nf-κB p65 in MDS-L cells treated with 2 μM GNA for the indicated hours. Band intensity was analyzed and normalized to that of β-actin by image lab and expressed as fold change to the level of untreated cells. (c) Activity of nf-κB reporter gene in parental or p65 KO MDS-L cells transfected with an nf-κB-sensitive luciferase reporter plasmid and treated with 2 μM GNA for 12 h before measurement of luciferase activity. The activity is expressed as fold change to the level of untreated cells. (d) Phosphorylation (Ser176/180) of IKKα/β in MDS-L cells treated with 2 μM GNA for the indicated times. Band intensity was analyzed and normalized to that of β-actin by image lab and expressed as fold change to the level of untreated cells. (e) Protein levels of nf-κB p65 in p65 KO MDS-L cells determined by using an anti-nf-κB p65 antibody. (f) Caspase 3/7 values of control, p65 KO and Fas KO MDS-L cells treated with or not treated with 2 μM GNA for 24 h. The activity is expressed as fold change to the level of untreated cells. (g) Interaction of nf-κB with microtubules. Whole cell lysates prepared from MDS-L cells were immunoprecipitated with anti-α-tubulin, IgG and GAPDH antibodies. The immunoprecipitates were resolved by SDS-PAGE and analyzed by Western blotting with antibodies against anti-α-tubulin, IgG and GAPDH, and the co-immunoprecipitated nf-κB in each sample was analyzed by Western blotting with an anti-nf-κB p65 antibody. Data are shown as means ± SD (*n* = 3). * *p* < .05, ** *p* < .01 and *** *p* < .001; ns: not-significant (one-way ANOVA followed by Tukey’s multiple comparison test).

It has been reported that microtubule-targeting agents activate the NF-κB signaling pathway in spite of cell death.^24,29–31^ NF-κB is activated by sequential cytoplasmic phosphorylation, ubiquitination, and degradation of the inhibitory protein IκB, which is associated with NF-κB, and subsequent nuclear translocation and posttranslational modifications, such as phosphorylation and acetylation, of NF-κB.^32–35^ Thus, the phosphorylation and degradation of the major IκB protein IκBα and phosphorylation of the NF-κB subunit p65 (RelA) in GNA-treated cells were assessed by Western blotting. Phosphorylation of IκBα increased with incubation time in GNA treatment with a concomitant decrease in the level of IκBα (Figure 7a). GNA also increased phosphorylation of p65, which peaked at 2 h after the stimulation (Figure 7b). Moreover, GNA induced activity of the NF-κB reporter gene that consists of NF-κB response element and luciferase (Figure 7c). IκBα is phosphorylated with IκB kinase (IKK) complex, which is composed of IKKα, IKKβ and IKKγ/Nemo and is activated by phosphorylation of IKKα/β.^36^ GNA increased phosphorylation of IKKα/β with incubation times of up to 40 min (Figure 7d). We then constructed p65 knockout (KO) MDS-L cells, in which null p65 protein was detected (Figure 7e), to examine whether the NF-κB signaling is involved in GNA-induced apoptosis of cells. GNA did not increase either the NF-κB reporter activity or caspase 3/7 activity in p65 KO MDS-L cells, in contrast to their stimulation in parental MDS-L cells (Figure 7c,f). Therefore, NF-κB activated by GNA plays an essential role in GNA-induced apoptosis of MDS-L cells.

There have been reports that show an association of NF-κB with microtubules.^24,37^ Immunoprecipitation experiments with an anti-tubulin antibody were performed to determine whether this is the case in MDS-L cells. The band of p65 was detected in immunoprecipitates of the cell extract with the anti-tubulin antibody but not in immunoprecipitates with either the anti-GAPDH antibody or IgG (Figure 7g).

### Involvement of fas expression via activation of the NF-κB signaling pathway in GNA-induced apoptosis

**Figure 8.**
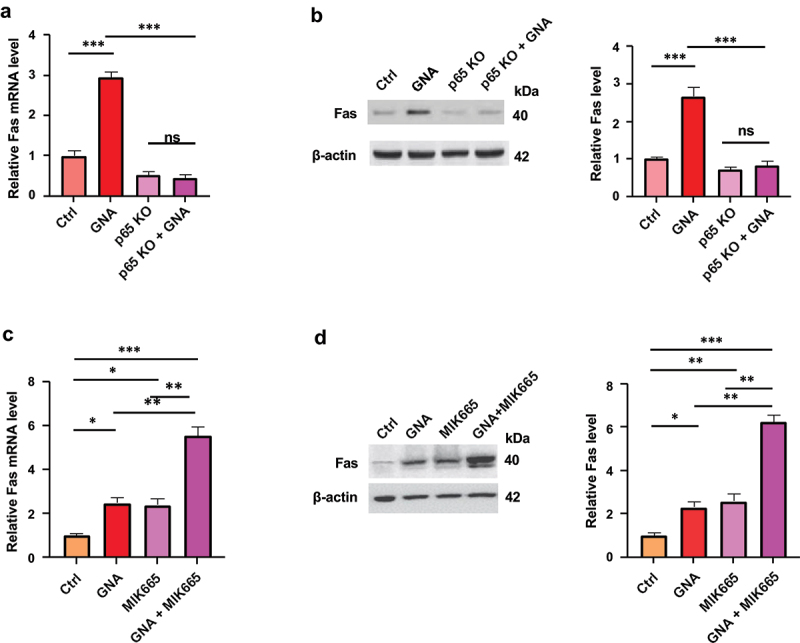
Upregulation of fas expression in GNA- and GNA plus MIK665-treated MDS-L cells. (a) Fas mRNA expression in gna-treated cells. Control and p65 KO MDS-L cells were treated with 4 μM GNA for 12 h. mRNA was extracted and converted to cDNA before RT-PCR analysis. Relative gene expression was calculated by the 2^−ΔΔCt^ method with β-actin as a housekeeping gene and expressed as fold change to the level of untreated cells. (b) Fas protein levels of control and p65 KO MDS-L cells treated with 2 μM GNA for 24 h. Band intensity was analyzed and normalized to that of β-actin by image lab and expressed as fold change to the level of untreated cells. (c) Fas mRNA expression in GNA plus MIK665-treated cells. MDS-L cells were treated with 4 μM GNA, 50 nM MIK665 or both for 24 h. Relative gene expression was expressed as fold change to the level of untreated cells. (d) Fas protein levels of GNA plus MIK665-treated cells. MDS-L cells were treated with 4 μM GNA, 50 nM MIK665 or both for 24 h. Band intensity was expressed as fold change to the level of untreated cells. Data are shown as means ± SD (*n* = 3). * *p* < .05, ** *p* < .01 and *** *p* < .001; ns: not-significant (one-way ANOVA followed by Tukey’s multiple comparison test).

Since it has been shown that NF-κB is a direct activator of Fas transcription,^38^ we measured Fas expression in GNA-treated cells to determine whether this is the case with GNA in MDS-L cells. Both the mRNA and protein levels of Fas significantly increased in GNA-treated parental cells, but GNA-induced upregulation of Fas was completely abolished in p65 KO MDS-L cells (Figure 8a,b). Moreover, the GNA-induced activation of caspase 3/7 was fully attenuated in Fas KO MDS-L cells (Figure 7f). The results indicate that GNA induces MDS cell apoptosis through upregulation of Fas expression mediated by activation of the NF-κB signaling pathway. Fas mRNA and protein expressions were also upregulated by MIK665, and the combination of GNA and MIK665 had synergistic effects on them (Figure 8c,d).

### Inhibition of MDS cell growth in an MDS xenograft model by GNA

**Figure 9.**
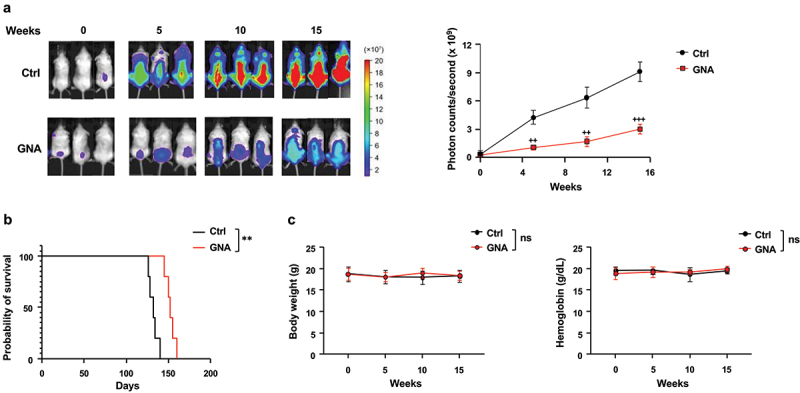
GNA inhibits the growth of mds‐L/Akaluc cells in an MDS model xenograft. NOG hIL-3 GM-CSF tg mice irradiated at a dose of 1.8 gy were injected with 1 × 10^7^ MDS-L/Akaluc cells via the tail vein. Four weeks after transplantation, recipient mice (*n* = 5 in each group) received intraperitoneal injections of a vehicle or 16 mg/kg GNA twice a week for 15 weeks. (a) Images of bioluminescence signals of proliferating MDS-L/Akaluc cells in representative mice (3 mice each) at 0, 5, 10 and 15 weeks after the start of treatment (left). Quantification of photon counts from mds‐L/Akaluc cells in MDS xenograft mice (right). Data are shown as means ± SD. (b) Kaplan – Meier survival curve of mice. Survival was evaluated from the starting point of treatment to death. (c) Body weight and blood hemoglobin levels of mice. Data are shown as means ± SD. Survival curves were analyzed by the Kaplan-Meier method and compared using the log-rank test (** *p* < .01). ^++^*p* < .01 and ^+++^*p* < .001 vs. control; ns: not-significant (student’s t-test).

We also evaluated the efficacy of GNA in a xenograft mouse model of MDS. In this in vivo analysis, we took advantage of the AkaBLI system, which is composed of Akaluc and AkaLumine-HCl and produces a brighter emission by a factor of 100 to 1,000 over the conventional BLI system,^39^ allowing us to monitor tumor burden more precisely. MDS-L cells were transduced with the Akaluc gene using a recombinant mScarlet-P2A-Akaluc lentivirus and selected by mScarlet expression as a marker. MDS-L/Akaluc cells were intravenously inoculated into NOG hIL-3 GM-CSF Tg mice after irradiation at a dose of 1.8 Gy.^40^ The tumor burden was monitored by bioluminescence signals. MDS-L cells with MDS-like features had a slow expansion in NOG hIL-3 GM-CSF Tg mice and induced lethal disease after a long latency. Recipient mice had been treated with GNA for 15 weeks. GNA significantly inhibited MDS-L cell growth and prolonged overall survival of the recipient mice (Figure 9a,b). Body weight and hemoglobin levels in the control and GNA-treated mice did not change significantly during the chemotherapy (Figure 9c). Thus, the effectiveness of GNA with a minimum side effect was observed in the xenograft model.

## Discussion

The results presented in this report indicated for the first time that GNA induced apoptosis of MDS-L cells along with inhibition of tubulin polymerization, activation of NF-κB through phosphorylation of IKKα/β and IκBα, and upregulation of Fas. Since the apoptosis was fully attenuated in either p65 KO or Fas KO cells (Figure 7f), NF-κB and Fas play crucial roles in GNA-induced apoptosis. The upregulation of Fas caused by GNA was completely abolished in p65 KO MDS-L cells, indicating NF-κB-dependent induction of Fas. Thus, these results obtained by using KO cells demonstrate that GNA induces MDS-L cell apoptosis through upregulation of Fas expression mediated by activation of the NF-κB signaling pathway. In contrast to this, it has been shown that Fas is not involved in the apoptosis of the HSC-3 human oral squamous cell carcinoma cell line induced by the microtubule-stabilizing agent docetaxel.^41^ Although the mechanism underlying the participation of NF-κB in Fas upregulation is not elucidated in this report, our results are consistent with the results of Liu et al. showing direct regulation of Fas transcription by NF-κB in human colon carcinoma cells and in mouse embryo fibroblasts.^38^ Alternatively, it is possible that NF-κB-dependent secretion of cytokines such as TNF-α and IFN-γ, which regulate Fas expression, contributes to the NF-κB-mediated induction of Fas.^42^

NF-κB is a transcriptional factor that regulates a number of genes involved in a broad range of biological processes including inflammation, cell proliferation, differentiation, and metastasis.^43,44^ It has also been shown to participate in the regulation of apoptosis of cells in response to various stimuli. The activation of NF-κB generally antagonizes apoptosis,^45^ but it has been reported that some stimuli and agents induce cancer cell apoptosis by activating NF-κB.^46,47^ Whether the activation of NF-κB antagonizes or promotes apoptosis has been shown to depend on the type of stimuli.^48^ Microtubule-targeting agents have been suggested to belong to the latter group,^24,29–31^ and our results showing that GNA caused inhibition of tubulin polymerization and NF-κB activation, which led to cell apoptosis, coincide with that notion. Although the opposing agents, which stabilize and destabilize microtubules, activate NF-κB, this effect can be related to their interference with the dynamic behavior of microtubules (combination of growth, shrinkage and rapid transitions between the two).^24^ The mechanism responsible for the activation of NF-κB caused by microtubule-targeting agents through inhibition of microtubule dynamics is not known. However, there are reports concerning the association of NF-κB with microtubules that we confirmed here (Figure 5f).^24,37^ It has been shown that NF-κB indirectly interacts with microtubules by its association with the complex of IκB and the outer-arm light-chain of dynein, a motor protein associated with microtubules.^49^ It is possible that binding of microtubule-targeting agents to microtubules or inhibition of microtubules assembly affects the association of NF-κB and microtubules through rearrangement of microtubules and thereby influences subsequent modification and/or degradation of IκB and/or translocation of NF-κB. In fact, it has been reported that the association of NF-κB with microtubules is enhanced by KN16 treatment of cells.^24^ Another possible factor to impact on the association of NF-κB and microtubules is acetylation of microtubules, which has been reported to be upregulated by microtubule-targeting agents and GNA (Figure 5b).^23,24,50^ The main acetylation residue of α-tubulin is Lys40, which localizes in the lumen of polymerized cylindrical microtubules.^51^ Although acetylation of microtubules occurs usually after microtubule assembly and is related to their stabilization or stability,^51,52^ it is upregulated by both the microtubule stabilizing and destabilizing agents mentioned above. This fact suggests that inhibition of microtubule dynamics is involved in the upregulation of microtubule acetylation and that microtubule acetylation has other functions besides contribution to stabilization or stability of microtubules. The acetylation neutralizes the positive charge of the lysine residue and can thus induce conformational change of the protein and alter the interaction with other proteins.^53^ It is interesting that the acetylated tubulin dimer associates with and inhibits plasma membrane Na^+^/K^+^ ATPase,^54^ because dynein motor protein has ATPase activity, which generates force and movement on microtubules.^55^ Further studies are necessary to determine whether and how the association of NF-κB with microtubules and acetylation of microtubules are involved in the activation of NF-κB caused by microtubule-targeting agents.

Although several reports have shown that GNA induces G0/G1 phase arrest in cancer cells,^13^ there are no GNA reports that indicate G2/M cell-cycle arrest of cells. Our results showed a significant increase of G2/M phase arrest in GNA-treated cells, but its increase was smaller than that of G0/G1 phase arrest of cells. This may be one reason why GNA-induced G2/M cell-cycle arrest has not been reported before. Microtubule-targeting agents interrupt the mitosis of cells by perturbing the microtubule dynamics.^56^ The GNA-induced increase in soluble tubulin along with the decrease in polymerized tubulin suggests that GNA inhibits microtubule assembly via binding to microtubules like most of the microtubule-destabilizing agents^57^ or affecting expression and/or activity of microtubule-binding proteins including those categorized as stabilizers and destabilizers^25,26^ and thereby interferes with progression of the M phase of the cell cycle. In fact, expression of stathmin, one of destabilizers, and the levels of its phosphorylated form with reduced activity increased and decreased, respectively, by GNA treatment in our study. Furthermore, GNA upregulated phosphorylation of the stabilizer MAP4 that reduces its activity. All of these changes may be at least partially responsible for GNA-caused inhibition of microtubule polymerization. As binding sites for microtubule-targeting agents on tubulin of microtubules, seven sites and domains have been identified: taxane, colchicine, laulimalide/peloruside, pironetin, and gatorbulin sites and vinca and maytansine domains.^57,58^ G2/M cell-cycle arrest is frequently followed by apoptosis, but less or no apoptosis is occasionally observed.^59,60^ Involvement of GNA-induced G2/M arrest in the apoptosis of MDS-L cells is not known. In this connection, it is noteworthy that GNA-induced apoptosis was fully dependent on Fas through the NF-κB signaling pathway.

It should be noted that GNA-induced apoptosis and growth inhibition of MDS cells including patients’ primary cells were accompanied by downregulation of MCL-1 expression and potentiated by the MCL-1 inhibitor MIK665 with synergistic induction of Fas because this gene is highly expressed in hematological malignancies and many types of solid tumors^61^ and because MCL-1 inhibitors are being developed as anticancer drugs. MCL-1 is an essential gene for cancer survival and tumorigenesis. A high expression level of MCL-1 in cancer cells changes the balance of proapoptotic and antiapoptotic proteins, which prevents cancer cells from programmed apoptosis, leading to malignant cell proliferation.^62^ Therefore, developing MCL-1 inhibitors could be a potential therapeutic approach for cancer treatment. In recent years, considerable progress has been made in the development of potent and highly selective MCL-1 inhibitors. Several compounds have been used in clinical trials, and the results suggest they are capable of inducing tumor cell apoptosis in preclinical settings. MIK665 is an MCL-1 inhibitor that has been used in phase 1 clinical trials for evaluation of the safety, tolerability, and anti-cancer activity of MIK665 for treatment of refractory or relapsed lymphoma or multiple myeloma (NCT02992483),^63^ and for treatment of AML and MDS (NCT02979366).^64^ Our results indicated a significant therapeutic effect of GNA when combined with the MCL-1 inhibitor MIK665 in vitro, suggesting that this combination regimen might be a promising therapeutic strategy for MDS treatment.

## Conclusion

GNA interfered with polymerization of tubulin in MDS cells, resulting in cell cycle arrest at the G2/M phase. GNA induced apoptosis of MDS cells, which was accompanied by a reduction in expression of the anti-apoptotic protein MCL-1, through upregulation of Fas expression mediated by the NF-κB signaling pathway, and inhibited their growth in vitro and in vivo with a minimum side effect. The anti-proliferative effect of GNA was potentiated by an MLC-1 inhibitor, suggesting that this combination regimen might be a promising therapeutic strategy for MDS treatment.

## Materials and methods

### Cell lines

MDS-L (Dr. Kaoru Tohyama, Kawasaki Medical University, Kurashiki, Japan) is a subline established from the human MDS cell line MDS92 obtained from the bone marrow of an MDS patient.^16,17^ This cell line has complex karyotypic abnormalities such as del(5q) [der(5)(5;19)], monosomy 7, *HIST1H3C* mutation (histone H3 K27M) and somatic mutations in *NRAS* and *TP53*.^16^ SKM-1 (DSMZ, ACC547) is a cell line established from an MDS/AML patient with no chromosomal abnormalities, but it has point mutations in *NRAS* and *KRAS*.^18^ TF-1 (ATCC, CRL-2003) is an immortal cell line derived from human erythroleukemia.^19^ Freshly isolated primary MDS cells (MDS-EB1 and MDS overt AML) were obtained as CD34^+^ cells from bone marrow (BM) aspirates of one patient with MDS and another patient with MDS/AML by isolation of BM-mononuclear cells (MNCs) using Ficoll-Paque (Sigma-Aldrich, product # GE17-5442-02), followed by isolation of CD34^+^ cells from BM-MNCs using a CD34 MicroBead kit (Miltenyi Biotec). The clinical values of the two patients are summarized in Table 1. All patients provided written informed consent according to institutional guidelines (Affiliated Hospital of Jining Medical University). The present study was approved by the Institutional Review Board of Jining Medical University (approval #20220613103). Human CB CD34^+^ cells (Cat #70008.5) were purchased from StemCell Technologies. The cell lines were routinely tested to verify the absence of mycoplasma contamination and underwent authentication through short tandem repeat DNA typing. All cells used were cultivated for less than 20 passages.

**Table 1.** Clinical values of patients whose primary MDS cells were obtained.

Patient	Patient 1	Patient 2
Sample	BM	BM
Diagnosis	MDS overt AML	MDS-EB1
Sex	Male	Male
Age	71	66
Karyotype	Normal	Normal
Blast (%)	48.9	3.1
Hb (g/dL)	8.2	6.6
WBC (/μL)	1830	320
MCV (fL)	86.8	85.3
PLT (×10^4^/μL)	2.7	2.2
WT1 mRNA (copy/μg RNA)	768	1145
R-IPSS	6	3.9

Freshly isolated primary MDS cells were obtained from BM aspirates of one patient with MDS and another patient with MDS/AML.

Abbreviations: Hb, hemoglobin; MCV, mean corpuscular volume; MDS-EB1, MDS with excess blast 1; PLT, platelet; R-IPSS, Revised International Prognostic Scoring System; WBC, white blood cell; WT1; Wilms’ tumor 1.

### Cell culture and drug treatment

MDS-L cells were cultured in RPMI-1640 containing human IL-3 (10 ng/mL) (BioLegend) and 10% fetal bovine serum (FBS, BioLegend). SKM-1 cells were cultured in RPMI-1640 with 20% FBS. TF-1 cells were cultured in RPMI-1640 containing GM-CSF (1 ng/mL) and 10% FBS. MDS overt AML and MDS-EB1 cells (Table 1) were cultured in RPMI-1640 containing stem cell factor (SCF) (10 ng/mL), GM-CSF (1 ng/mL), IL-3 (10 ng/mL), thrombopoietin (TPO) (10 ng/mL), fms related receptor tyrosine kinase 3 (FLT3) ligand (10 ng/mL) and 20% FBS. Penicillin and streptomycin were added to all of the above culture media at the final concentrations of 100 IU/mL and 100 μg/mL, respectively. Those cell lines were incubated in a humidified atmosphere containing 5% CO_2_ at 37°C. Human CB CD34^+^ cells were cultured in serum-free StemSpan Animal Component-Free (ACF) media (StemCell Technologies) supplemented with 1% antibiotic/antimycotic (Life Technologies) and standard growth factors (SGF) consisting of 50 ng/ml SCF, 50 ng/mL TPO, 80 ng/mL FLT3 ligand and 100 ng/mL IL-6 (Miltenyi Biotec), at 37°C with 5% CO_2_ and 5% O_2_. A stock of GNA (Selleckchem #S9031; batch number: S903104; purity: 99.94%) was prepared in DMSO (Sigma-Aldrich) and diluted with a complete medium before use. For growth assays, cells were seeded on 24-well plates at 1 × 10^5^ cells/mL in triplicate and treated with graded concentrations of GNA. Cell proliferation was assessed by counting the viable cells or by using an MTS assay kit (Promega).

### Apoptosis and cell cycle assays

Apoptosis was assessed by using the Caspase-Glo^Ⓡ^ 3/7 Assay (Promega) according to the manufacturer’s instructions. Flow cytometric analysis of the cell cycle was performed on BD FACSCelesta^TM^ (BD Biosciences) by the use of the BrdU Flow Kit (BD Biosciences).

### Construction of gene KO cells

MDS-L cells were seeded in a 6-well tissue culture plate (3 × 10^5^ cells/well). The cells were transfected with CRISPR/Cas9 knockout (KO) plasmids (NF-κB p65: sc -400,004-ko-2; Fas: sc -400,481-KO-2; control: sc -418,922 from Santa Cruz). Each of the KO plasmids carries a different guide RNA specific for the target gene, as well as the Cas- and GFP-coding regions. GFP-positive cells were selected by cell sorting 48 h after transfection. Depletion of the target proteins was confirmed by Western blotting.

### AkaBLI system

In vivo bioluminescence imaging (BLI) is a noninvasive method for measuring light output produced by the enzyme-catalyzed oxidation reaction of a substrate. The AkaBLI system, composed of Akaluc enzyme and AkaLumine-HCl as a high permeability substrate, provides a light source of sufficient strength to penetrate body walls, even in deep tissue areas.^39^ pcDNA3/Venus-Akaluc was obtained from the RIKEN BioResource Research Center (Catalog No: RDB15781). The nucleotide sequence of the synthetic construct Akaluc gene for the firefly luciferase mutant protein Akaluc is available under DDBJ accession No: LC320664. Regarding the generation of the CS-CDF-UbC-mScarlet-P2A-Akaluc-PRE lentiviral vector, mScarlet (synthesized by FASMAC Co., Ltd., Japan) and Akaluc CDS were cloned into the CS-CDF-UbCG-PRE lentiviral vector (Catalog No: RDB08363, a gift from Dr. H. Miyoshi) downstream of the ubiquitin C promoter (UbC) using the AgeI and XhoI restriction sites, replacing the existing GFP CDS. Cloning primers were designed to include an in-frame addition of the GSG-P2A self-cleaving peptide sequence between the mScarlet and Akaluc sequences.

### Lentiviral production and transduction

A recombinant mScarlet-P2A-Akaluc lentivirus (LV) was generated by transient co-transfection of HEK293T cells with CS-CDF-UbC-mScarlet-2A-Akaluc-PRE and the helper plasmids pMD2.G (Addgene #12259) and psPAX2 (Addgene #12260) using the polyethylenimine method. Culture supernatants were collected after 96 h and filtered (0.22 µm), followed by the concentration of LV particles by centrifugation at 40,000 g for 4 h.

### Xenograft studies

NOD.Cg-Prkdc^scid^ Il2rg^tm1Sug^ Tg (SRa-IL3, CSF2)/Jic (NOG hIL-3 GM-CSF Tg) mice expressing human IL-3 and GM-CSF were purchased from the Central Institute for Experimental Animals (Kawasaki, Japan).^40^ All mice were housed under specific pathogen-free conditions of 25 ± 1◦C temperature, 50–60% relative humidity and a 12-h light/dark cycle, and allowed free access to sterilized water and solid feed. MDS-L/Akaluc cells (1 × 10^7^ cells) were inoculated into female NOG hIL-3 GM-CSF Tg mice that had been irradiated at a dose of 1.8 Gy. Four weeks after tumor inoculation, the mice were randomly divided into two groups and treated with DMSO or GNA, which was diluted with saline, twice a week for 15 weeks through intraperitoneal injection. One hundred microliters of 5 mm AkaLumine-HCl (Wako) was injected intraperitoneally into mice just before the imaging analysis, and mice were imaged under isoflurane anesthesia within 5 to 10 min after the injection. The following conditions were used for image acquisition: open for total bioluminescence, exposure time = 60 sec, binning = 4 to 8, field of view = 25 × 25 cm, and f/stop = 1. In vivo photon counting was conducted with an IVIS system using Living Image 2.5 software (Xenogen). Mice were monitored until they became moribund, at which time they were euthanized. Hemoglobin was assessed by an automated hematology analyzer (Beckman Coulter LH 780).

### Immunoblot analysis of tubulin in MDS cells

MDS-L cells were cultured in the presence of GNA for 6 h. The cells were washed with phosphate-buffered saline, permeabilized with 200 μL of pre-warmed buffer (80 mm PIPES-KOH, pH 6.8, 1 mm MgCl_2_, 1 mm EGTA, 0.2% Triton X-100, 10% glycerol, and 1 × protease inhibitor), and incubated at 30°C for 5 min. Supernatants containing the soluble fraction of tubulin were separated after centrifugation, mixed with 4 × Laemmli gel sample buffer, and boiled for 3 min. To collect the insoluble polymerized tubulin fraction, 250 μL of 1 × Laemmli gel sample buffer was added to the pellet, followed by boiling for 3 min. The fractions containing soluble and polymerized tubulin were separated by SDS-PAGE, transferred to an Immobilon-P transfer membrane (Millipore) and probed with a mouse anti-human α-tubulin antibody followed by incubation with an HRP-linked anti-mouse IgG (Invitrogen, #31430). Immunodetection was performed using the Clarity ECL Western Blotting Substrates kit (BIO-RAD, Cat #1705061).

### Western blotting

Tumor cells were lysed in lysis buffer (20 mm Tris-HCl, pH 7.4, 150 mm NaCl, 1 mm EDTA, 1% Triton X-100) plus a protease inhibitor cocktail (Abcam, ab271306) for 60 min on ice. Proteins were separated on a 4–20% SDS-polyacrylamide gradient gel and transferred to an Immobilon-P transfer membrane (Millipore). The following antibodies were used after dilution with TBS containing 0.1% Tween 20 and 3% nonfat dry milk: anti-p65 (Abcam, ab16502), anti-phospho-p65 (Ser536) (CST, #3031), anti-IκBα (Abcam, ab97783), anti-IκBα phospho-Ser32 (CST, #9241), anti-Fas (Invitrogen, PA5–115214), anti-MCL-1 (CST, #4572), anti-BCL-2 (Abcam, ab59348), anti-BCL-6 (Abcam, ab19011), anti-BAX (Abcam, ab216494), anti-α-tubulin-acetyl-Lys40 (CST, #3971), anti-α-tubulin (Invitrogen, MA1–19162), anti-stathmin 1 (Abcam, ab131481), anti-phospho-stathmin 1 (S63) (Abcam, ab192648), anti-MAP4 (Bethyl Laboratories, #A301-489A), anti-phospho-MAP4 (S787) (GL Biochem), anti-IKKα + anti-IKKβ (GeneTex, GTX52348), anti-phospho-IKKα/β (Ser176/180) (CST, #2697), and anti-β-actin (Sigma-Aldrich, A5441) followed by incubation with an HRP-linked anti-rabbit IgG (CST, #7074) or anti-mouse IgG (Invitrogen, #31430). Immunodetection was performed using the Clarity ECL Western Blotting Substrates kit (BIO-RAD). For a co-immunoprecipitation experiment, MDS-L cells were treated with GNA, paclitaxel, and TN16 for 16 h at the indicated doses. After treatment, the cells were treated with 0.2% Triton X-100 for 5 min and then washed twice with phosphate-buffered saline. Whole cell lysates were prepared, and immunoprecipitation was performed with α-tubulin, IgG and GAPDH antibodies using equal amounts of proteins. After immunoprecipitation, Western blotting was performed using antibodies specific for α-tubulin, GAPDH, and IgG, and the co-immunoprecipitated NF-κB was detected using an anti-p65 antibody. Quantifications were done using ImageJ software.

### Nf-κB reporter assay

For NF-κB reporter activity experiments, MDS-L or p65 KO cells were transfected with a plasmid in which three copies of an Igκ-derived NF-κB response element sits in a basal IL-2 promoter and directs the transcription of luciferase from pGL3 (Promega). Cells were harvested 24 h after GNA treatment. The medium was removed from the cultures, and 200 μL of Passive Lysis Buffer (Promega) was added per well. The plate was allowed to sit for 15 min and then put into a − 80°C freezer. The plate was thawed the next day, and the cells were scraped into Eppendorf tubes and vortexed and then spun to remove debris. The supernatant was assayed by mixing 5 μL of the test solution with 95 μL of luciferase substrate buffer (Promega) and light emission was read on a Turner Designs TD-20/20 luminometer.

### RT-qPCR analysis

Total RNA from MDS-L cells was extracted using the RNeasy Micro Kit according to the manufacturer’s protocol (Qiagen). cDNA was synthesized by reverse transcription using 50 ng total RNA, reverse transcriptase, RNAsin, and dNTPs (all Promega). For gene expression, real-time quantitative polymerase chain reaction (RT-qPCR) was performed on a CFX96 and CFX384 Real-Time PCR System (BioRad) using the SYBR-Green technology. Relative gene expression was calculated by the 2^−ΔΔCt^ method with β-actin as a housekeeping gene. The sequences of primers for FAS were as follows: forward, 5’-GGACCCAGAATACCAAGTGCAG-3’; reverse, 5’-GTTGCTGGTGAGTGTGCATTCC-3’. The sequences of primers for β-actin were: forward, 5’-CACCATTGGCAATGAGCGGTTC-3’; reverse, 5’-AGGTCTTTGCGGATGTCCACGT-3’.

### Statistical analysis

Data are shown as the mean ± SD. In statistical analyses, p-values were derived using unpaired Student’s t-tests for any studies with only two groups. Otherwise, comparisons of groups were performed on log-transformed data using a one-way ANOVA test followed by Tukey’s multiple comparison test. Survival curves were calculated by the Kaplan-Meier method and compared using the log-rank test. All analyses were conducted using GraphPad Prism Software.

## Data Availability

The datasets used and analyzed during the current study are available from the corresponding author, [C.Z.], on reasonable request.

## Abbreviations

7-AAD: 7-aminoactinomycin D
ALLO-HSCT: allogeneic hematopoietic stem cell transplantation
AML: acute myeloid leukemia
BLI: bioluminescence imaging
BrdU: bromodeoxyuridine
CB: cord blood
FBS: fetal bovine serum
FLT3: fms related receptor tyrosine kinase 3
GNA: gambogenic acid
HMA: hypomethylating agent
KO: knockout
LV: lentivirus
MDS: myelodysplastic syndrome
SCF: stem cell factor
TPO: thrombopoietin
